# Discovery of a kleptoplastic ‘dinotom’ dinoflagellate and the unique nuclear dynamics of converting kleptoplastids to permanent plastids

**DOI:** 10.1038/s41598-019-46852-y

**Published:** 2019-07-19

**Authors:** Norico Yamada, John J. Bolton, Rosa Trobajo, David G. Mann, Przemysław Dąbek, Andrzej Witkowski, Ryo Onuma, Takeo Horiguchi, Peter G. Kroth

**Affiliations:** 1 Department of Biology, University of Konstanz, Konstanz, Baden-Württemberg 78457 Germany; 2 Department of Biological Sciences, University of Cape Town, Cape Town, Western Cape 7701 South Africa; 3 Marine and Continental Waters Program, Institute for Food and Agricultural Research and Technology, Sant Carles de la Ràpita, Catalonia, 43540 Spain; 4 Royal Botanic Garden Edinburgh, Edinburgh, Scotland EH5 3LR United Kingdom; 5 Institute of Marine and Coastal Sciences, University of Szczecin, Szczecin, West Pomerania 70383 Poland; 6 Department of Gene Function and Phenomics, National Institute of Genetics, Mishima, Sizuoka 4118540 Japan; 7 Department of Biological Sciences, Hokkaido University, Sapporo, Hokkaido 0600810 Japan

**Keywords:** Evolutionary developmental biology, Cellular microbiology, Chloroplasts

## Abstract

A monophyletic group of dinoflagellates, called ‘dinotoms’, are known to possess evolutionarily intermediate plastids derived from diatoms. The diatoms maintain their nuclei, mitochondria, and the endoplasmic reticulum in addition with their plastids, while it has been observed that the host dinoflagellates retain the diatoms permanently by controlling diatom karyokinesis. Previously, we showed that dinotoms have repeatedly replaced their diatoms. Here, we show the process of replacements is at two different evolutionary stages in two closely related dinotoms, *Durinskia capensis* and *D. kwazulunatalensis*. We clarify that *D. capensis* is a kleptoplastic protist keeping its diatoms temporarily, only for two months. On the other hand, *D. kwazulunatalensis* is able to keep several diatoms permanently and exhibits unique dynamics to maintain the diatom nuclei: the nuclei change their morphologies into a complex string-shape alongside the plastids during interphase and these string-shaped nuclei then condense into multiple round nuclei when the host divides. These dynamics have been observed in other dinotoms that possess permanent diatoms, while they have never been observed in any other eukaryotes. We suggest that the establishment of this unique mechanism might be a critical step for dinotoms to be able to convert kleptoplastids into permanent plastids.

## Introduction

It is now widely accepted that almost all eukaryotic plastids originated from a free-living cyanobacterium, which was taken up by a heterotrophic eukaryote^1,2^. This event, known as the primary endosymbiosis, occurred about 1.6 to one billion years ago^3^. Direct descendants possessing primary plastids are classified into the supergroup Archaeplastida, which consists of three extant lineages, glaucophytes, rhodophytes (red algae), and chloroplastida (green algae and land plants)^4,5^. However, the acquisition of phototrophy is not an event unique to Archaeplastida. It has been revealed that other endosymbiosis events have occurred independently many times in different eukaryotic groups^1,2,6,7^. The plastids are called secondary plastids or higher order plastids (e.g. tertiary or beyond) because their heterotrophic ancestors engulfed microalgae which possessed primary or higher order plastids^1,2^.

Dinoflagellates are a group of such eukaryotes, which have been assumed to have acquired their plastids via secondary endosymbiosis^1,2,5^. However, in fact, only approximately half of the dinoflagellates have maintained their photosynthetic ability with their secondary plastids, called peridinin-type plastids, which were originally derived from a red alga^8,9^. Most other dinoflagellates have lost their photosynthetic ability and regained a fully heterotrophic mode of nutrition, independently in a number of different dinoflagellate taxa^9–11^. However, a few dinoflagellates belong to neither of the two main groups. Instead, they possess a variety of different kinds of plastids, differing in their origins from different microalgae and in the degree of integration into the host. For instance, all cryptomonad-derived plastids in dinoflagellates^12,13^, the haptophyte-derived plastids in the dinoflagellate *Phalacroma mitra*^14^ and those in an undescribed Antarctic dinoflagellate of the family Kareniaceae^15^ are kept temporarily as kleptoplastids. Kleptoplastic species ‘steal’ organelles, including plastids, from microalgae or from other kleptoplastic protists for performing photosynthesis^16,17^. There are two types of kleptoplasty. In one the kleptoplastic protists selectively retain only ingested plastids^18,19^. In the other the protists maintain some of the other ingested prey organelles as well as the plastids^20^, especially mitochondria and nuclei^16^. Johnson and his co-authors have shown in the kleptoplastic ciliate *Mesodinium rubrum* that the prey nucleus keeps its transcriptional activity, and have therefore named this type of kleptoplasty ‘karyoklepty’^16^. However, because of a lack of synchronous cell division between host and the ingested organelles, or due to digestion of the ingested organelles, kleptoplastic protists lose the kleptoplastids and have to feed on free-living microalgae or protists^21^. In contrast to these kleptoplastic species, the haptophyte-derived plastids of all members of the family Kareniaceae^22^, except for the undescribed Antarctic dinoflagellate mentioned above^15^, are established as permanent genuine organelles of host dinoflagellates and so are equivalent to the peridinin-type plastids. This is true also of the green alga-derived plastids in *Lepidodinium* spp.^23^. This plastidial diversity of dinoflagellates is evidently due to both independent replacements of peridinin-type plastids by other microalgae, and by the uptake of microalgae as new plastids by heterotrophic dinoflagellates^10,22–24^.

The monophyletic dinoflagellates known as ‘dinotoms’^25^, classified in the family Kryptoperidiniaceae^26^, recruit diatoms as their tertiary plastids^24^. The diatoms retain their own nuclei, mitochondria and the endoplasmic reticulum in addition to their plastids, and these diatom organelles are separated by a single membrane from the cytosol of their host dinoflagellates^27–29^. Transcriptome analysis of two dinotoms, *Durinskia baltica* (strain CS-38) and *Kryptoperidinium foliaceum* (strain CCMP1326), has revealed that almost no functional reduction has occurred in the diatom nuclei of both dinotoms^30^. Although these dinotom studies have demonstrated that the diatoms of dinotoms keep the autonomy structurally and genetically from their hosts, it has been observed that host dinoflagellates are able to maintain them permanently by controlling karyokinesis^31,32^. Based on these ultrastructural and genetic assessments, it has been suggested the diatoms of dinotoms are an evolutionary intermediate stage of plastids, between kleptoplastids and genuine plastids^2,33^. In fact, the diatoms in dinotoms are not strictly endosymbionts^7^, because they lack silica frustules^27–29^, but here we use the word ‘endosymbiotic’ to describe them, in accordance with previous studies of dinotoms.

**Figure 1:**
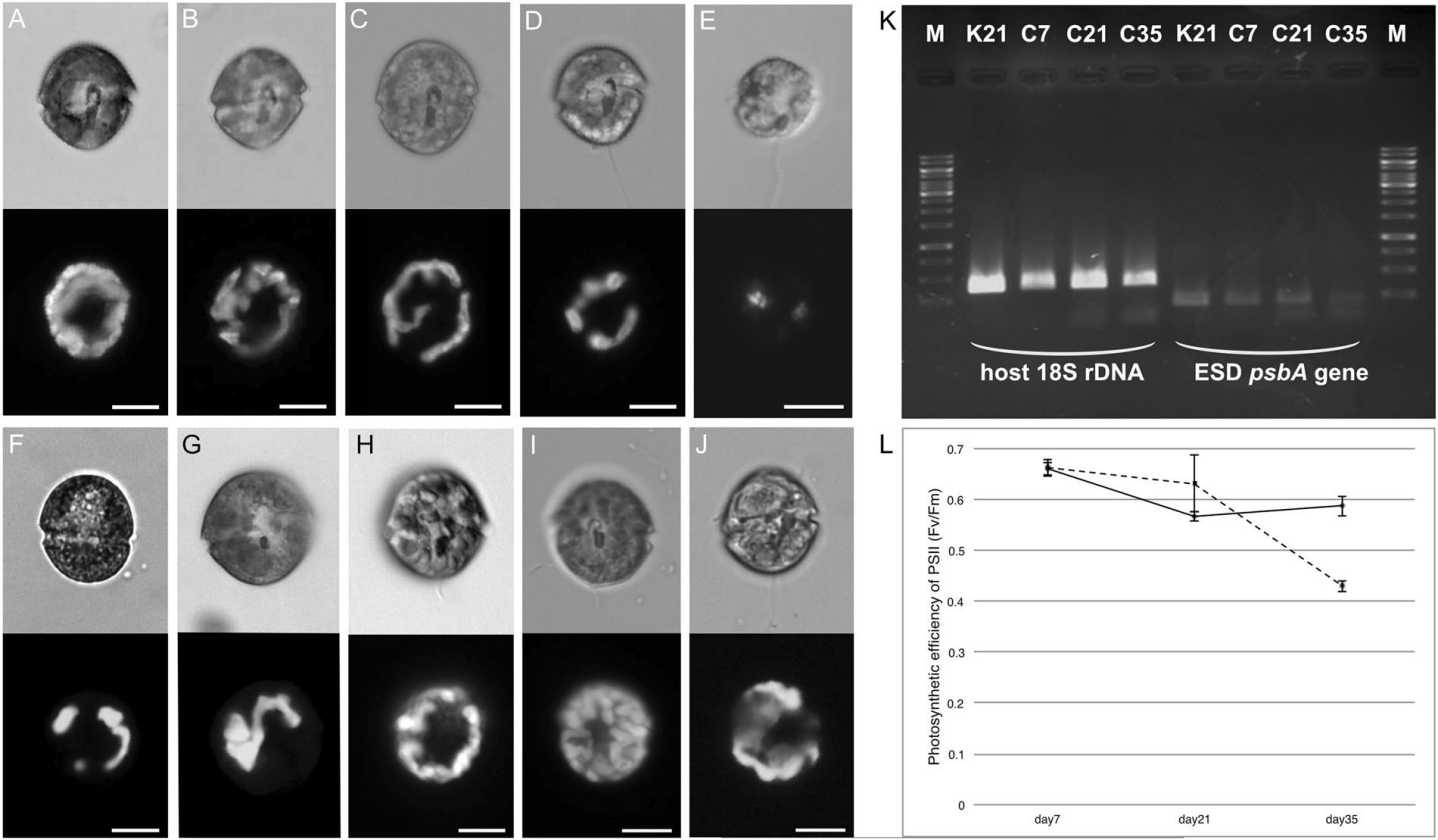
Plastid dynamics in *Durinskia capensis* and *D. kwazulunatalensis* with light and fluorescence microscopy (**A**–**J**), with semi-quantification of cDNA (**K**), and with PAM (**L**). (**A**–**E**) *D. capensis* cells cultured on days 7 (**A**), 21 (**B**), 35 (**C**), 49 (**D**), and 63 (**E**) respectively under autotrophic conditions. (**F**–**H**) *D. capensis* cells co-cultured for 63 days with free-living *Nitzschia inconspicua* (**F**), *N*. cf. *agnita* (**G**) or *Nitzschia* sp. (**H**). (**I**,**J**) *D. kwazulunatalensis* cells cultured on days 21 (**I**), and 63 (**J**) under autotrophic conditions. Upper row = Ventral view by light. Lower row = Ventral view by chlorophyll *a* autofluorescence. Scale bar = 10 µm. (**K**) The PCR assay of cDNA from *D. capensis* and *D. kwazulunatalensis*. The nucleus-encoded 18 S rDNA of *D. capensis* strongly expresses from days 7 to 35, while there is an obvious drop in the expression of the plastid-encoded *psbA* gene by day 35. C = *D. capensis*, K = *D. kwazulunatalensis*, M = Marker (GeneRuler 1 kb DNA Ladder, Thermo Scientific, Waltham). Number indicates cultured periods under autotrophic conditions. The gel-image is uncropped. The RNA extractions of these samples were processed in parallel in each culturing day, and then cDNA syntheses from these RNAs and the PCR amplifications were processed together. (**L**) Time-dependent observation of the maximum quantum yield of PSII (Fv/Fm) of *D. capensis* and *D. kwazulunatalensis* under autotrophic conditions. *D. kwazulunatalensis* (smooth line) is able to maintain the photosynthetic activity of PSII in day 7 to 35, while that of *D. capensis* (dash line) reduces the value at day 35.

Recently it was revealed that each dinotom species possesses phylogenetically distinct endosymbiotic diatoms (ESDs), except for *Galeidinium*-related spp. and two *Unruhdinium* spp. (Supplementary Data Table S1)^34^. On current knowledge, at least twelve ESD replacements have occurred in dinotoms, and as a result, fourteen different diatom species, belonging to six genera, are utilised as endosymbionts by nineteen dinoflagellate host species (Supplementary Data Table S1)^34–36^. Even though the fact of the frequent ESD replacements indicates that the degrees of integration of the ESDs are not the same in all dinotoms, it has been assumed so far that all dinotoms have established permanent relationships with the ESDs^31,32^. Here, we show that two closely related dinotoms, *Durinskia capensis* and *D. kwazulunatalensis* possess different stages in the evolution of ESDs. The former has not yet established a permanent relationship and gradually loses the ESDs (Fig. 1). Our molecular phylogeny showed that *D. capensis* utilises two closely related diatom species in nature, one of which corresponds morphologically to *Nitzschia agnita* (Supplementary Data Figs S1A and S2). In contrast, *D. kwazulunatalensis* is able to retain multiple ESDs permanently without any diatom predation^34^. Its permanent ESDs are all derived from a diatom of the genus *Simonsenia* (Supplementary Data Fig. S1A). To confirm that *D. capensis* is a kleptoplastic dinotom, and to investigate what drove the difference in endosymbiotic levels in these closely related dinotoms, we compared the time-dependent behaviours of their ESDs microscopically, molecular biologically and physiologically, and conducted feeding experiments on *D. capensis* using four free-living pennate diatoms.

## Results

### Dynamics of the diatom plastids in *Durinskia capensis* and *D. kwazulunatalensis*

Since there are no available cultured strains of *Durinskia capensis*, all experiments in this study were performed using cells isolated directly from field samples collected in the type locality of *D. capensis*, rocky tidal pools at Kommetjie, Western Cape, South Africa^37^. The collected dinoflagellates were identified as *D. capensis* based on their morphology as well as 18 S rDNA molecular phylogeny on the initial sampling (Supplementary Data Fig. S1B). All subsequent cells from the same location were identified by the morphology alone. The ESD of *D. capensis* used in this study was closely related but a different species from the type ESD of *D. capensis*^37^. These two ESDs formed a clade with 100% bootstrap support (Supplementary Data Fig. S1A), while the *rbc*L gene sequences of these two diatom species differs by 1.08% (1382 bp, 110 bp at the 5′ end and 22 bp in the middle part of the type sequence were lacking). Each set of 50 *D. capensis* cells, isolated from the field seawater samples, was cultured under autotrophic, osmotrophic (with soil extract or dissolved carbon sources), or phagotrophic conditions with one of four *Nitzschia sensu lato* diatoms collected in South Africa: *N. inconspicua* (strain IRTA-CC-1), *N*. cf. *agnita* (strain IRTA-CC-152), *Nitzschia* sp. (strain SZCZP1124) or *Psammodictyon* sp. (strain SZCZP1020) (Supplementary Data Figs S1A and S2). In particular, our molecular phylogeny showed that *N*. cf. *agnita* is the exactly same species of the type ESD of *D. capensis* (Supplementary Data Fig. S1A).

**Figure 2:**
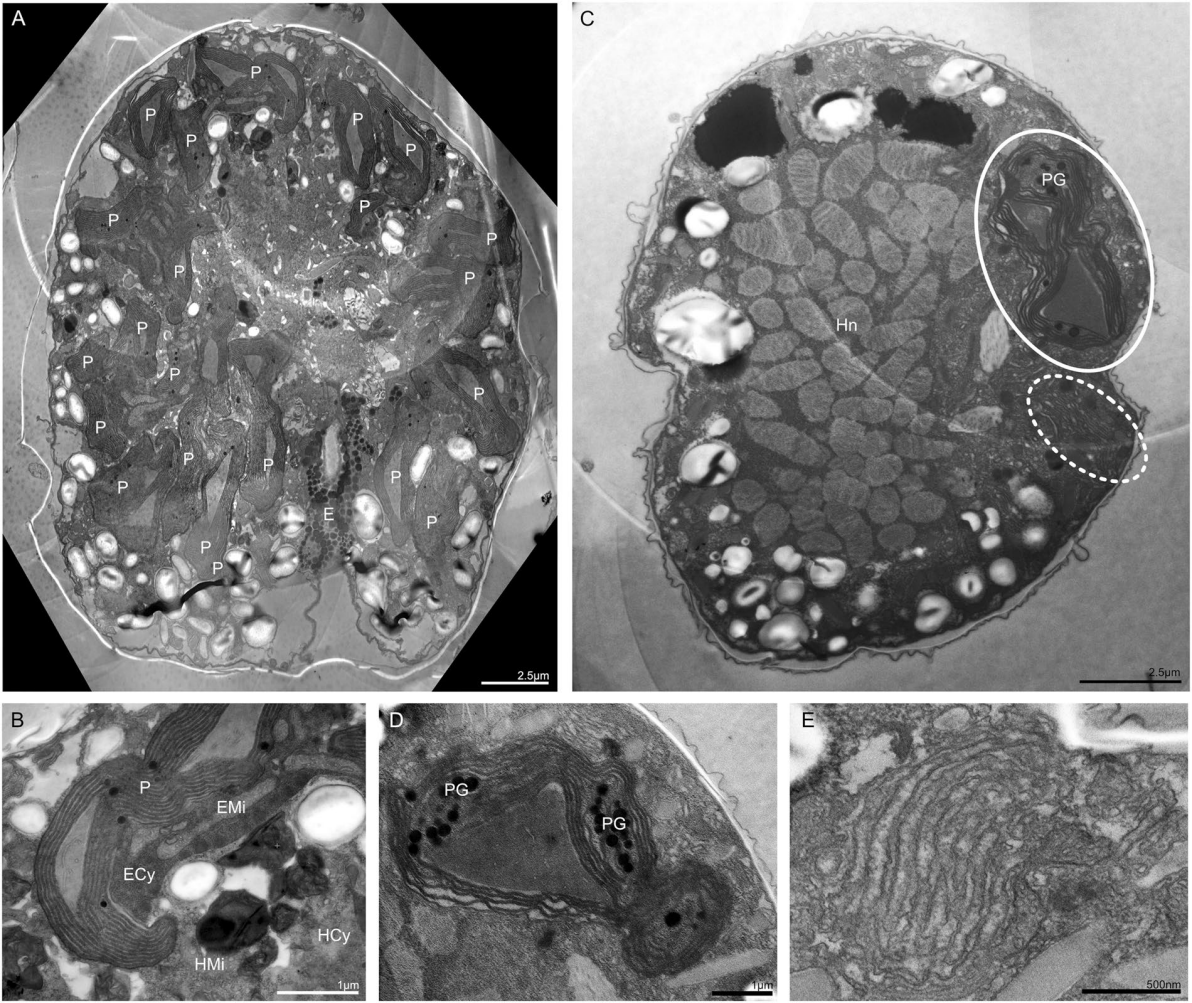
Plastid dynamics in *Durinskia capensis* observed with TEM under autotrophic conditions. (**A**) A single cell of *D. capensis* on day 7 maintains many healthy plastids (P). E = Eyespot. (**B**) Healthy plastids (P) and diatom mitochondria (EMi) are surrounded by the diatom cytoplasm (ECy) on day 7. These diatom organelles are clearly separated from the dinoflagellate cytoplasm (HCy). HMi = Dinoflagellate mitochondrion. (**C**) A single cell of *D. capensis* on day 35 contains two types of degraded plastids. The smooth ellipse indicates an active plastid, which contains senescence products, plastoglobuli (PG). The dotted ellipse indicates an inactive plastid-like structure with disrupted thylakoid-like structures. Hn = Dinoflagellate nucleus. (**D**) An active plastid in day 35, which contains plastoglobuli. Parts of thylakoids containing plastoglobuli are starting to lose their structure. The diatom cytoplasm and mitochondria were not observed. (**E**) An inactive plastid-like structure in day 35 with disrupted thylakoid-like structures.

Autotrophic and osmotrophic conditions gave the same results: on day 7 after collection, the plastids were widely distributed below the cell surface (Fig. 1A) and the mean length of the host cells was 22.8 μm (±1.93, n = 5). Subsequently, however, the plastids of the ESDs began to shrink gradually and by day 63 after collection, the *D. capensis* cells had lost most of their chlorophyll *a* autofluorescence, and the mean cell length had reduced to 13.4 μm (±0.66, n = 4, Fig. 1E). Transmission electron microscopy (TEM), cDNA semi-quantification and photosynthetic efficiency measurements showed that the diatom plastids gradually decomposed within the *D. capensis* cell. A single cell at day 7 contained a lot of healthy plastids (Fig. 2A,B), while at day 35, it contained two types of plastids (Fig. 2C): healthy plastids starting to be digested (Fig. 2D), and senescent plastid-like structures with disorganised thylakoid-like structures (Fig. 2E). Semi-quantification via PCR of cDNA showed that the expression of the plastid-coding *psbA* gene from the ESD in *D. capensis* dropped at day 35 (Fig. 1K). The photosynthetic activity by pulse amplitude modulation chlorophyll fluorometer (PAM) also showed the maximum quantum yield of PSII (Fv/Fm) of *D.capensis* reduced over a month (Fig. 1L).

**Figure 3:**
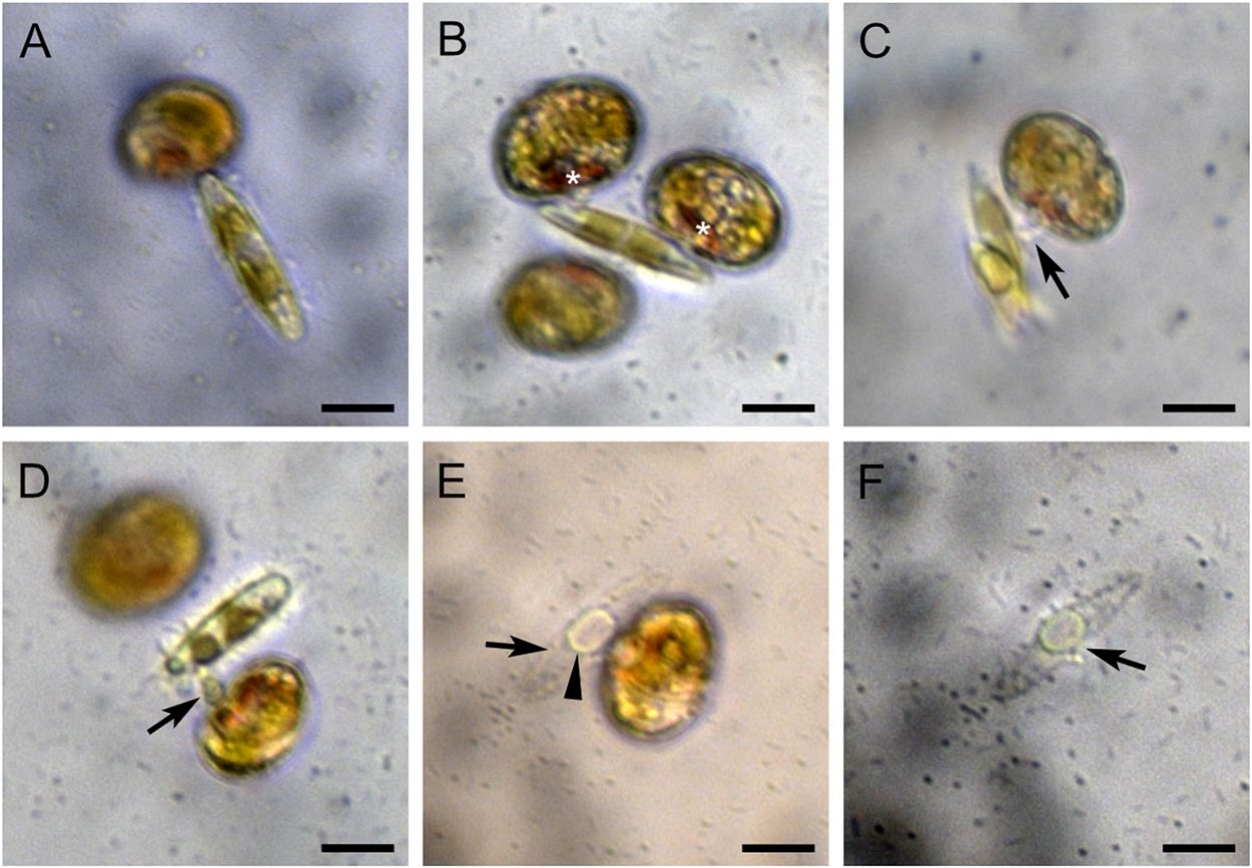
Diatom *Nitzschia* cf. *agnita* (strain IRTA-CC-152) are taken up by *Durinskia capensis*. (**A**) A *D. capensis* cell attacks the marginal part of a *N*. cf. *agnita* cell. (**B**) Two *D. capensis* cells attach to a *N*. cf. *agnita* cell by their ventral sides. The red particles (asterisks) are eyespots of *D. capensis*, located on the ventral side. (**C**) A *D. capensis* has impaled a *N*. cf. *agnita* cell by the peduncle (arrow). (**D**) Diatom organelles are absorbed via myzocytosis using a peduncle (arrow). (**E**) The diatom frustule (arrow) and the plasma membrane (arrowhead) are not ingested by *D. capensis*. (**F**) After *D. capensis* left, the broken part of the frustule (arrow) can be observed. Scale bar = 10 µm.

In contrast to the behaviour in autotrophic and osmotrophic conditions, the plastid and cell sizes remained stable after culturing, even up to day 63, when *D. capensis* was co-cultured with *N. inconspicua* (Fig. 1F), *N*. cf. *agnita* (Fig. 1G), or *Nitzschia* sp. (Fig. 1H). In particular, when *N*. cf. *agnita* was added to the crude cultures of *D. capensis* the dinoflagellate showed feeding behaviour within 30 minutes, attached to the diatom cells with its ventral side (Fig. 3A,B), and absorbed the diatom organelles using a peduncle (a feeding tube of dinoflagellates) by myzocytosis (Fig. 3C,D); it left behind the diatom frustule and a small amount of the diatom cytoplasm (Fig. 3E,F). On the other hand, *Psammodictyon* sp. was not effective in maintaining *D. capensis*, and all *D. capensis* cells disappeared by day 35*. D. kwazulunatalensis* could maintain the plastid and cell sizes, and the Fv/Fm without any diatom present (Fig. 1I,J,L).

### Dynamics of the diatom nuclei in *Durinskia capensis* and  *D. kwazulunatalensis*

**Figure 4:**
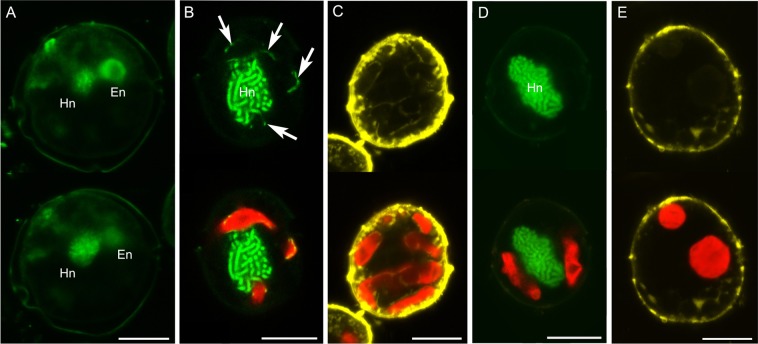
Diatom nuclear dynamics in *Durinskia capensis* observed with CLSM under autotrophic conditions. SYBR-Green or DAPI stained nucleus = Green, Mitotracker Green stained mitochondria = Yellow, Chlorophyll *a* autofluorescence = Red. (**A**) *D. capensis* ethanol-fixed on the sampling day (day 0). There are two round nuclei, a host dinoflagellate nucleus (Hn), which contains condensed chromosomes, and a diatom nucleus (En). (**B**) By day 7, the round diatom nucleus has disappeared from the *D. capensis* cell; instead string-shaped DNA signals can be observed alongside the plastids (arrow). (**C**) The Mitotracker Green-stained cell on day 7 indicates that the string-shaped DNA signals are from diatom mitochondria. (**D**,**E**) On day 35, no DNA signals from the diatom organelles can be observed in *D. capensis* with SYBR-Green (**D**) nor with Mitotracker Green (**E**). Sclae bar = 10 µm.

**Figure 5:**
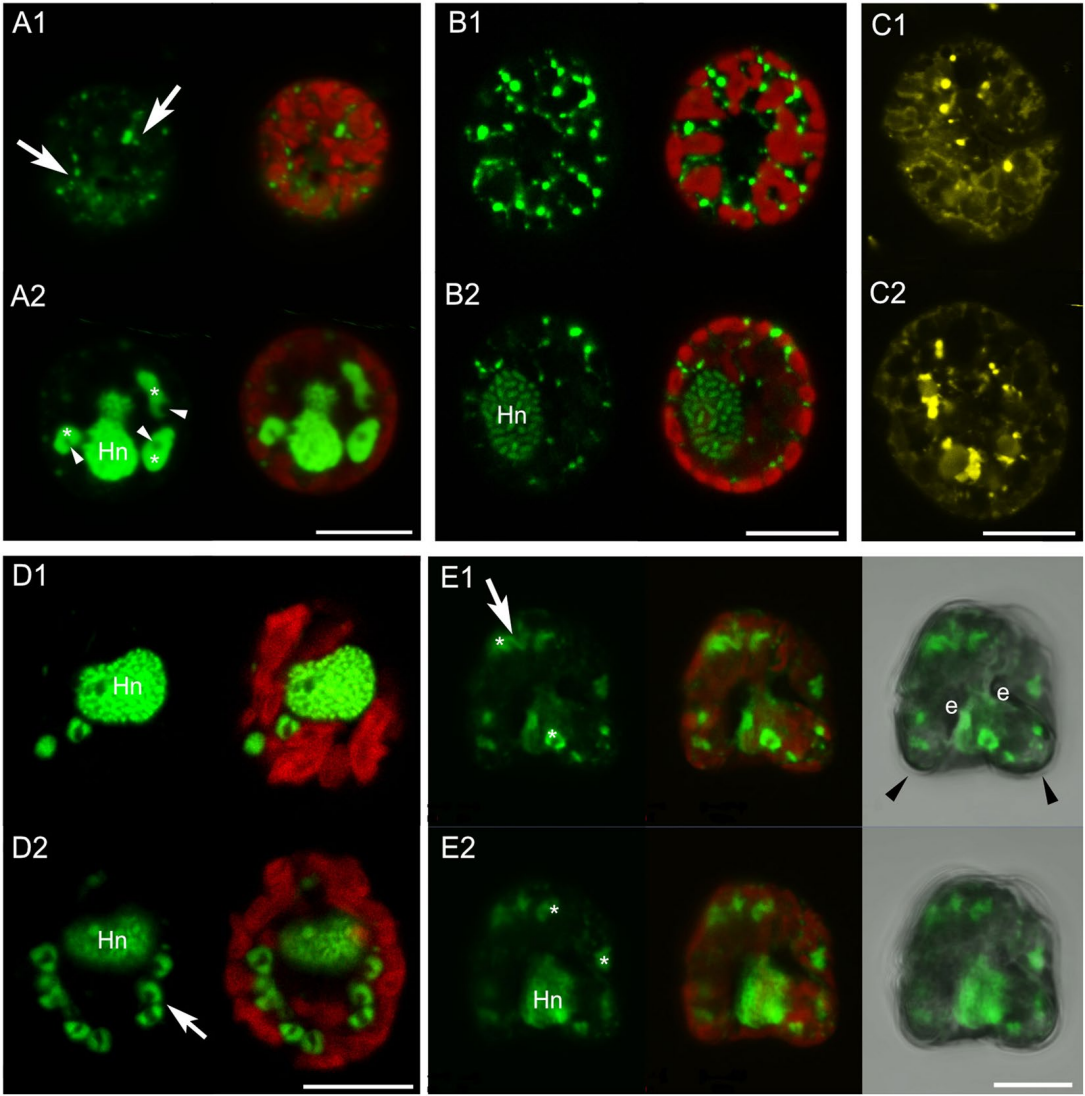
Diatom nuclear dynamics in *Durinskia kwazulunatalensis* observed with CLSM. SYBR-Green stained nucleus = Green, MitoTracker Green stained mitochondria = Yellow, Chlorophyll *a* autofluorescence = Red. (1) = peripheral sections of cells, (2) = the middle sections of cells. (**A**) A cell directly after cell division. Three condensed diatom nuclei (asterisks) and string/dot-shaped nuclei (arrows) can be seen in addition to the host dinoflagellate nucleus (Hn). The round dark parts in the diatom nuclei (arrowheads) are nucleoli. (**B**) A cell in host interphase. The condensed diatom nuclei have disappeared, leaving only the string/dot-shaped complexes of diatom nuclei dispersed at the cell periphery, especially near or between the plastids. (**C**) A cell in host interphase. Mesh-shaped mitochondria, probably diatom mitochondria, are observed in the periphery of the cell (C1), while large dot-shaped mitochondria, probably dinoflagellate mitochondria, are located in the middle of the cell (C2). (**D**) A cell prior to cell division. The diatom nuclei are all condensed. Arrow indicates a replicated pair of diatom nuclei. (**E**) Two daughter cells forming during host cell division. Daughter cells (arrowhead) share a host dinoflagellate nucleus (Hn). Several condensed diatom nuclei (asterisks) separate into daughter cells alongside the plastids and begin decondensing into string/dot-shaped structures (arrows). e = eyespot. Scale bar = 10 µm.

**Figure 6:**
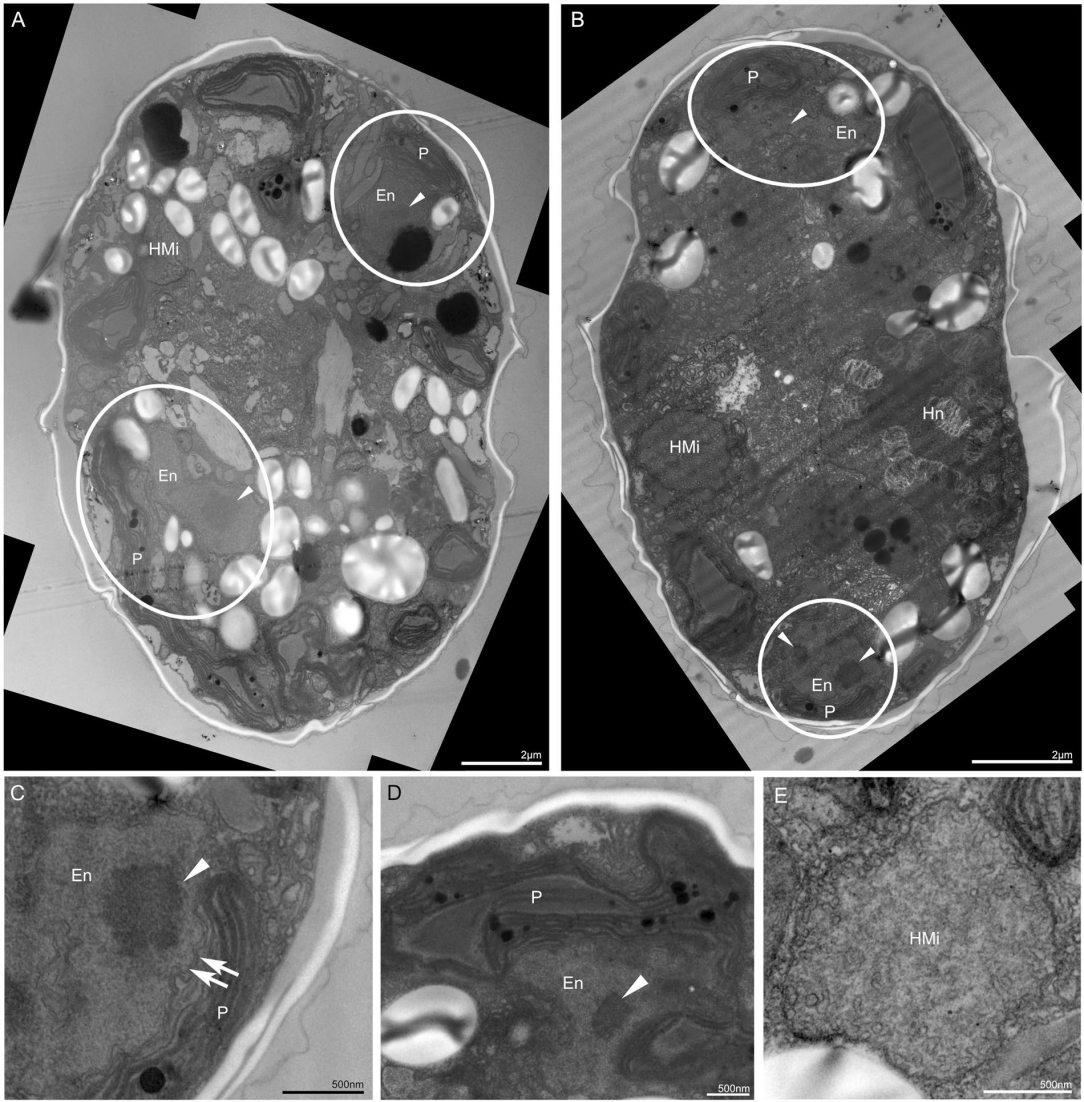
Diatom nuclear localizations during the interphase of *Durinskia kwazulunatalensis* with TEM. (**A**,**B**) Two diatom nuclei (En) on each individual cell are observed in the periphery of cells (circles). HMi = Dinoflagellate mitochondrion, Hn = Dinoflagellate nucleus. Arrowhead = Diatom nucleolus. (**C**,**D**) Each diatom nucleus, containing a nucleolus (arrowheads), is located alongside the plastids. Arrow = Nuclear membrane. (**E**) Giant dinoflagellate mitochondria (HMi) are often observed. P = Plastid.

Confocal laser scanning microscopy (CLSM) and TEM showed that *Durinskia capensis* ingested the diatom nucleus with the plastids but lost it rapidly in culture. Thus, in samples fixed on the day of collection (day 0), *D. capensis* cells had two round nuclei (Fig. 4A), as mentioned in the original description of *D. capensis*^37^. One nucleus was that of the dinoflagellate while a round nucleus without condensed chromosomes might be the engulfed nucleus of a diatom (Fig. 4A). By day 7, there was already no sign of a diatom nucleus (Fig. 4B), but because all diatom nuclei in *D. capensis* disappeared during shipping from the type locality to Germany, we could not determine exactly when the diatom nucleus disappeared. On day 7, we observed string-shaped DNA signals alongside the plastids instead of a round diatom nucleus (Fig. 4B). CLSM and TEM samples indicated these were composed of diatom mitochondrial DNA (Figs 2B and 4C). The diatom mitochondria also disappeared by day 35 from the *D. capensis* cell, under both CLSM (Fig. 4D, E) and TEM (Fig. 2C,D). In contrast, *D. kwazulunatalensis* exhibited unique morphological dynamics for the ESD nuclei. Unlike other dinotoms, *D. kwazulunatalensis* contains three to six round ESD nuclei in a single cell (Fig. 5A, Supplementary Data Fig. S3A). During the host interphase, these multiple ESD nuclei decondensed into a string or dot-like complex close to, or between, the plastids (Figs 5B and 6A–D, Supplementary Data Fig. S3B). Mesh-shaped diatom mitochondria were also located near the cell surface. They could be distinguished from decondensed diatom nuclei by their structure (Fig. 5B and C-1), and from host dinoflagellate mitochondria by their location and size (Figs 5C-2 and 6B,E). Before the host cell divided, the string or dot-shaped nuclei changed back into three to six rounded nuclei, each of which replicated independently but simultaneously (Fig. 5D, Supplementary Data Fig. S3C). After division, the round nuclei separated to the host daughter cells (Fig. 5E, Supplementary Data Fig. S3D), initially remaining compact in daughter host cells after division (Fig. 5A, Supplementary Data Fig. S3A), but soon dispersing back into their diffuse interphase state.

## Discussion

Our experiments show clearly that *Durinskia capensis* is a kleptoplastic dinotom, the first one ever described. In the absence of free-living diatoms, *D. capensis* plastids gradually decompose over two months, and the transcriptional activity of the plastid-encoded gene and the photosynthetic activity reduce over a month, while the engulfed diatom nucleus disappears rapidly by day 7. The engulfed diatom mitochondria also disappear by day 35. In contrast, when this same dinoflagellate is co-cultured with particular free-living diatoms it is able to maintain the plastid and cell sizes. *D. capensis* shows a moderate preference for diatoms: three *Nitzschia* spp. are utilised as kleptoplastids, whereas *Psammodictyon* sp. is ineffective in maintaining the *D. capensis* cells and all *D. capensis* were dead by day 35. *D. capensis* shows particularly active feeding behaviour when *N*. cf. *agnita* is added to the crude cultures.

The existence of kleptoplastic dinotoms has been implied in two preceding studies. The first example is a bloom-forming dinotom that was found in South Carolina, USA^38^. Although the dinotom was identified as *Kryptoperidinium foliaceum*, a species which has been widely studied and shown to be a dinotom possessing a permanent-ESD^29,30,32^, PCR assays and fluorescence microscopy of the South Carolina dinotom showed that it does not possess any diatom nucleus. The authors of this study suggested that the ESD in this *K. foliaceum* might be a temporary endosymbiont^38^. The second example is a bloom-forming freshwater dinotom, *Unruhdinium niei* (=*Peridiniopsis niei*)^26^ collected from Donghu Lake and Xiangxi River in China^39^. Comparative microscopical studies of two *Unruhdinium* species indicated that *U. niei* did not possess any diatom nucleus^39^. The authors concluded that *U. niei* had already lost the diatom nucleus, and could be regarded as a more advanced stage in the evolution of plastids, but it is also possible that this species is kleptoplastic, and loses the diatom nucleus rapidly like *D. capensis*. Thus, although these interpretations have not been verified under laboratory conditions, we speculate that there are other kleptoplastic dinotoms besides *D. capensis*. Molecular phylogenetic analyses have revealed that dinotoms have repeatedly replaced their ESDs with other diatoms, which must live in the same habitats as the host dinoflagellates^34,40,41^. We suggest that ‘dinotoms’ are a mixed lineage of dinoflagellates possessing a wide variety of diatom-derived plastids at different stages of integration with their host, due to the repeated ESD replacements.

In this study, we also succeeded in observing that *D. capensis* consumes diatoms using the feeding tube, a peduncle. Although it has been suggested that dinotoms have replaced the ESDs repeatedly^34,40,41^, the feeding behaviour of dinotoms has been unknown until this study. Our observation confirmed that dinotoms only ingest diatom organelles using the peduncle; the plasma membrane and the frustule of diatoms are not engulfed. This is the reason why diatom frustules have never been observed within dinotoms^27–29^. We suggest that a single membrane, which surrounds the diatom organelles, separating them from the host cytoplasm^27–29^, is not a diatom-derived membrane, and may be produced by dinoflagellates during the diatom feeding. Observing the peduncle also indicates that the ESDs in dinotoms are not defined ‘endosymbionts’^7,42^: prey diatoms are non-viable after they are consumed by dinoflagellates. Some ingested diatom organelles maintain their activities in the dinotoms for performing photosynthesis, but these diatom organelles cannot survive independently of dinotom cells. We infer that ‘ESDs’ in dinotoms have developed into permanently-retained plastids via kleptoplasty, but not via whole algal endosymbiosis^43^, and that they should be known as ‘organelle-retaining diatom-derived tertiary plastids (ODP)’ not ‘endosymbiotic diatoms (ESD)’^7,42^. This study shows, for the first time, experimental evidence that, in certain algal lineages, permanent plastids have evolved from kleptoplasty not from endosymbiosis.

Our results also indicate that *D. kwazulunatalensis* could be a suitable model organism for understanding the process of conversion of kleptoplastids into permanent plastids. *D. capensis* and *D. kwazulunatalensis* are closely related phylogenetically and morphologically. The difference between their 18 S rDNA sequences is only 0.8%. Two plates of the epitheca on their dorsal sides are slightly different morphologically^34^. Furthermore, their source localities are close geographically: both species occur in tidal pools in South Africa, *D. capensis* on the southwest coast, facing the Atlantic Ocean^37^, while *D. kwazulunatalensis* lives along the east coast, on the Indian Ocean^34^. Interestingly, this study revealed that single *D. kwazulunatalensis* cell possesses multiple diatom nuclei. Because all other dinotoms possess a single diatom nucleus^40,41,44–48^, we hypothesise that the ODPs in *D. kwazulunatalensis* are relatively younger permanent plastids, compared to the ODPs of other dinotoms. Some dinotoms may have gained multiple diatom nuclei during the kleptoplastic phase, but after the association between ODPs and the hosts had further evolved, the number of diatom nuclei may have been reduced to one. In fact, it has been observed that kleptoplastic species are able to maintain several microalgal nuclei at the same time, which have been taken up from different cells of the same prey species^49^.

Kleptoplastic organisms have been reported from several different eukaryotic lineages^7^. However, most of these kleptoplastic species do not have close relatives that have developed permanent plastid stages. The only exception is an undescribed Antarctic dinoflagellate, the Ross Sea Dinoflagellate (RSD), which possesses haptophyte-derived kleptoplastids^15^. A eukaryotic nucleus was observed in the RSD cell, in addition to the host dinoflagellate nucleus, although it has not been confirmed whether the additional nucleus is an engulfed haptophyte one^50^. Interestingly, the RSD belongs to the family Kareniaceae, in which all other members possess permanent haptophyte-derived plastids, while all of the other haptophyte organelles have already been lost^51^. The genetic differences in 18 S rDNA sequences between RSD and the other Kareniaceae are 1.7 to 2.4%^15^. Therefore, it is likely that the phylogenetic relationship between the RSD and other Kareniaceae is more distant, and thus the stages in the evolution of the plastids may differ more, when compared to the relationship between *D. capensis* and *D. kwazulunatalensis*. *D. kwazulunatalensis* is a ‘missing link’ microalga, illustrating an intermediate stage between other dinotoms possessing a permanent diatom nucleus and the kleptoplastic *D. capensis*.

**Figure 7:**
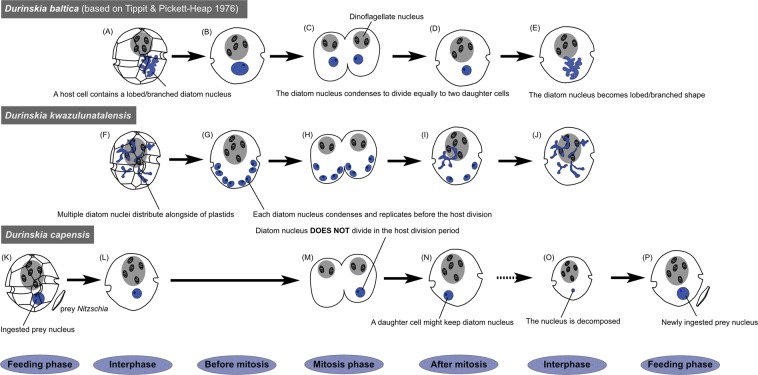
A scheme of diatom nuclear dynamics in three dinotoms, *Durinskia baltica* (strain LB1563)^31^*, D. kwazulunatalensis* and *D. capensis*. (**A**) A single diatom nucleus in *D. baltica* exhibits a lobed/branched-shape in interphase. (**B**) The diatom nucleus condenses and becomes rounded for replication, and then (**C**) divides equally, one entering each host daughter cell. (**D**) After cell division, the diatom nucleus initially remains round in the daughter cells, (**E**) but subsequently, returns to complex shape. (**F**) During interphase, multiple diatom nuclei in *D. kwazulunatalensis* are located alongside the plastids with a string/dot-shaped form. (**G**) Each diatom nucleus condenses and becomes round and each is capable of replication prior to host cell division. (**H**) The replicated condensed nuclei separate equally into the two daughter cells. (**I**) After division of the host, the diatom nuclei decondense again, (**J**) and finally disperse throughout the periphery of the cells near the plastids. (**K**) *D. capensis* ingests the diatom nucleus with the plastids. (**L**) A round diatom nucleus is observed after the ingestion. (**M**) The diatom nucleus probably does not replicate prior to the host cell division, as in other kleptoplastic species^20,49^, (**N**) we expect only one of the daughter cells inherits the diatom nucleus. (**O**) After a time, the inherited diatom nucleus might be decomposed, like the plastids. (**P**) *D. capensis* re-ingests a new free-living diatom as kleptoplastids.

Lastly, we would like to comment on the replication of diatom nuclei in *D. capensis* and *D. kwazulunatalensis*. Our CLSM observations indicate that in *D. capensis* the ingested diatom nucleus is unable to replicate (Fig. 7), because by culture day 7 none of the *D. capensis* cells possessed the diatom nucleus. Generally, kleptoplastic protists selectively retain only the ingested plastids and other prey organelles are transferred to the host digestive vacuole^19^, while in kleptoplastic protists exhibiting karyoklepty^16^, the ingested nucleus does not divide but is inherited by one of the daughter cells^20,49^. In this study, we could not directly observe the fate of the ingested diatom nucleus in *D. capensis* because of the delay between harvesting the natural population and the first cytological observations in our lab; nor could we determine whether the ingested diatom nucleus maintains transcriptional activity. To answer this question requires further molecular biological studies. On the other hand, *D. kwazulunatalensis* has already established a replication system for the multiple diatom nuclei present, which display unique morphological dynamics. During host interphase, the diatom nuclei are string/dot-like structures and disperse alongside the diatom plastids. These string-shaped nuclei then condense, become round, and replicate prior to host cell division. After host cell division, the round nuclei disperse and revert to string or dot shapes (Fig. 7). Similar behaviour of the permanent ODPs has been observed in two other dinotoms, *Durinskia baltica* (strain LB1563, Fig. 7)^31^ and *Kryptoperidinium foliaceum* (no strain number)^27^ or (strain Baiona A3, B1 and B9)^32^. Again, their diatom nuclei become lobed or branched during host interphase but condense into round bodies prior to host division. To our knowledge, such nuclear dynamics have never been reported for any other eukaryotes, including free-living diatoms^52^, kleptoplastic species^20,49^, and the nucleomorphs in cryptomonads^53^ or chlorarachniophytes^54^. These facts indicate that nuclear morphological dynamics are shared only among dinotoms possessing permanent ODPs. We suggest that the establishment of these nuclear dynamics may be critical for conversion of temporary ODPs to the permanent ODPs in dinotoms. Interestingly, dispersion and condensation of DNA have been reported in the plastidial and mitochondrial nucleoids of green algae and land plants^55,56^. In land plants, the plastidial nucleoids change in shape and localisation depending on the developmental stage of the plastids^57^, while in the green alga *Chlamydomonas reinhardtii* they vary during the cell cycle^55^. In land plants, thylakoid formation seems to be tightly linked with nucleoid morphology and distribution^58^. However, at present, we do not yet know whether these nucleoid dynamics are related to the diatom nuclear dynamics of dinotoms. Finding a kleptoplastic dinotom and its close relative with young permanent ODPs is a great opportunity to study the exact mechanism of these unique nuclear dynamics, to understand how hosts can control and maintain the early stages of plastids permanently.

## Methods

### Sample collections and cultures

Because of the lack of available *Durinskia capensis* cultures, field water samples were collected eight times (between December 2016 and October 2018) in the type locality of *D. capensis*, i.e., the rocky tidal pools at Kommetjie, Western Cape, South Africa^37^. Water samples were collected from the same tidal pool in each sampling. Due to transport time from South Africa to Germany, all experiments on *D. capensis* started from day 7 after collection, except for CLSM observations of samples fixed with ethanol (final ethanol concentration 70%) on the sampling day (day 0). *D. capensis* cells were isolated individually from the collected field water samples using capillary pipettes with two rinses in drops of sterilized medium, and then 50 cells were cultured in each of two sterilized autotrophic media, f/2^59^ or Daigo’s IMK (Nihon Pharmaceutical Co., Ltd., Tokyo), and four sterilized osmotrophic media (f/2 or IMK media with 1 g/l alpha-D-glucose, 1 g/l yeast extract and 1 g/l peptone, or with soil extract). *N. inconspicua* and *N*. cf. *agnita* were isolated from the same water samples as those collected for *D. capensis* in December 2017 and October 2018. Single cells were transferred using capillary pipettes into Petri dishes containing sterilized f/2 medium to establish clonal culture (i.e., strain IRTA-CC-1 and IRTA-CC-152).

Established strains of *D. kwazulunatalensis* (strain CX22, collected at Marina Beach, Kwazulu-Natal, South Africa), *Nitzschia* sp. (strain SZCZP1124, collected in Churchhaven, Western Cape, South Africa) and *Psammodictyon* sp. (strain SZCZP1020, collected from surf zone at Tsaarsbank, Western Cape, South Africa) were cultured in sterilized IMK medium (for CX22) or f/2 medium (for diatoms) without additional osmo/phagotrophic sources. All species were cultured at 20 °C, approximating the water temperature in the field at the time of sampling, with an illumination of 50 µmol·photons·m^−2^·s^−1^ under a 16:8 h light:dark cycle.

### Feeding experiment

Four diatom species of *Nitzschia sensu lato* were used in the feeding experiments, because they were collected from geographically close places to the *Durinskia capensis* type locality. To starve *D. capensis*, firstly 50 *D. capensis* cells were cultured in sterilized f/2 medium in each of three culture vessels for 10 days. Subsequently, approximately 50 to 100 cells of clones *N. inconspicua*, *N*. cf. *agnita*, *Nitzschia* sp. or *Psammodictyon* sp. were added to each culture. The feeding photos were taken with ZEISS AxioCam MRc (Zeiss, Oberkochen) under an inverted microscope, ZEISS Axiovert 40 CFL (Zeiss, Oberkochen).

### Light and fluorescence microscopy

For *Durinskia capensis*, five cells of each culture (on days 7, 21, 35, 49, 63, cultured in the absence of diatoms, and on day 63, cultured in the presence of *Nitzschia* spp.) were isolated individually using capillary pipettes. *D. kwazulunatalensis* cells on days 21 and 63 were collected by centrifugation at 3000 g. Both species were observed with an OLYMPUS BX51 microscope (Olympus, Tokyo); the camera was ZEISS AxioCam MRm (Zeiss, Oberkochen).

### Confocal laser scanning microscopy

For *Durinskia capensis*, five cells from each cultivation sample (on days 0, 7, 21 or 35) were isolated individually using capillary pipettes, and each was put in a 10 μl drop of IMK medium respectively. *D. kwazulunatalensis* on day 21 was centrifuged at 3000 g and 100 μl of IMK medium added to the pellet. For staining the nuclei of *Durinskia* spp., 500X working solution of SYBR Green I (Lonza, Basel, for living cells) or 125 μg/ml of DAPI (Carl Roth, Karlsruhe, for ethanol-fixed day 0 samples of *D. capensis*) were used for a final concentration of 3% (v/v) or 6% (v/v), respectively. All staining was developed over 20 minutes in darkness before observations. For staining mitochondria of *D. capensis* and *D. kwazulunatalensis*, 1 mM MitoTracker Green FM (Invitrogen, Carlsbad) was used at a final concentration of 100 nM. The sample was incubated for 90 minutes under the same conditions as used for culture of *D. capensis* and *D. kwazulunatalensis*. All observations were performed using a Zeiss LSM 700 (Zeiss, Oberkochen). To observe the diatom nucleus dynamics during the cell cycle, CLSM observations of *D. kwazulunatalensis* were performed early in the morning (04h00 for observing cells before division and dividing cells), mid-morning (09h00 for cells after division), or afternoon (13h00 for host interphase).

### Transmission electron microscopy

Two different protocols were used; a single cell-TEM protocol for *Durinskia capensis*, and a protocol for species established in culture for *D. kwazulunatalensis*.

*D. capensis*: On day 7 and 35, five *D. capensis* cells, cultured in the absence of diatoms, were isolated under the inverted microscope and were fixed for 30 min on ice in a cocktail of 1% (w/v) OsO_4_ and 2.5% (v/v) glutaraldehyde in 0.5 M sucrose made up in 0.5 M phosphate buffer (pH 7.1). The fixed cells were transferred, under the microscope, onto a 4% agarose-coated Nunc Thermanox coverslip (Thermo Fisher, Massachusetts) and left for 10 min to allow the cells to become immobilised on the agarose. Subsequently, the sample on the coverslip was rinsed once in the same buffer with 0.5 M sucrose and twice in buffer alone, keeping it in each solution for 10 min. The washed sample was post-fixed using 2% OsO_4_ made up in 0.5 M phosphate buffer for 1 hour on ice, then washed with 30% acetone twice, and then dehydrated for 10 min ascending concentrations of acetone (30%, 50%, 70%, 80%, 90%, and 95%). Lastly, it was dehydrated completely by washing twice for 30 min in 100% acetone. Infiltration of samples was then carried out with an acetone resin mixture. Spurr’s resin^60^ was prepared by mixing the component chemicals directly prior to use; 54.5 g of ERL 4206, 31.35 g of DER 736, 133.9 g of NSA, and 1.78 g of DMAE. 100% acetone and the Spurr’s resin were mixed in a 3:1, a 1:1, and a 1:3 ratio, and the sample was introduced sequentially to each higher resin concentration for 15 min. Finally, the cells were embedded in 100% resin for 30 min twice, then polymerized at 65 °C for 48 h and sectioned using a diamond knife on an ultramicrotome (LEICA EM UC6, Wetzlar). Picked samples were coated with carbon by evaporating a heated carbon rod in a Balzers BAF 301 apparatus (Balzers, Liechtenstein) under vacuum (<10^−4^mbar). The stream of carbon vapour to the sample was directed by an acceleration voltage of 2000V at 90 mA for 5–7 sec. Sections were viewed using a Zeiss EM912 Omega transmission electron microscope (Zeiss, Oberkochen).

*D. kwazulunatalensis*: Cells at day 21 after inoculation were harvested by centrifugation at 3000 g. After removing the supernatant, the sample was fixed for 30 min on ice with the same fixation solution of *D. capensis*, and then rinsed once in the same buffer with 0.5 M sucrose, and subsequently twice in buffer alone for 10 min each. The washed cells were post-fixed using 2% OsO_4_ made up in 0.5 M phosphate buffer for 1 hour on ice. The protocols after fixation were same to those of *D. capensis*.

### RNA extraction and cDNA amplification for quantifying gene-expression

Five dinoflagellate cells of *Durinskia capensis* were harvested by micropipette on each of days 7, 21 and 35, and of *D. kwazulunatalensis* on day 21 for RNA extraction, using the QuickExtract FFPE RNA Extraction Kit (Epicentre, Wisconsin). The QuickExtract FFPE solution was heated to 56 °C and maintained there for 30 min, followed by 98 °C for 2 min. The DNA was removed with the DNase I treatment provided with the kit. The extracted RNAs were used as a temple for cDNAs by High-Capacity cDNA Reverse Transcription Kits (Thermo Fisher Scientific, Massachusetts). Synthesised cDNAs were amplified with two primer-pair: SR1b + SR3 (for host nucleus-encoded 18 S rDNA)^34^, or DiatpsbAF (:AGGTATCTGGTTAACTGCTATG) + DiatpsbAR (: CTGGTAATACTTCACCTGAAGCTA) (for diatom plastid-encoded *psb*A gene), or SR8+ DiatSR12 (for diatom nuclear-encoded gene)^34^. The PCR protocols were as follows: an initial cycle at 95 °C for 3 min, followed by 40 cycles of the PCR steps: denaturation at 98 °C for 20 s, annealing at 52 °C for 15 s, and extension at 72 °C for 1 min. The final extension cycle was at 72 °C for 1 min. The amount of the gene expression was checked by electrophoresis gel. Because all diatom nuclei in *D. capensis* disappeared during shipping from the type locality to Germany, we could not get any diatom nuclear RNAs.

### Photosynthetic efficiency of ODPs

*Durinskia capensis* was co-cultured with *Nitzschia* cf. *agnita* (strain IRTA-CC-152) for three weeks. *N*. cf. *agnita* cells normally grew attached to the bottom of the culture vessel, whereas *D. capensis* liked to swim near the water surface, except during feeding. To prepare mono cultures of *D. capensis*, we collected only the supernatant of the *D. capensis* crude culture, then transferred it to fresh IMK culture medium, which contained 3 mg/l GeO_2_, which prevents diatom growth^61^. These mono dinoflagellate cultures were checked for the presence of *N*. cf. *agnita* under an inverted microscope, and it was confirmed that the cell numbers of this diatom were less than 5% of total dinoflagellate cell numbers. Strains of *D. kwazulunatalensis* were maintained without any diatom present. Samples of cultured dinoflagellates were taken on days 7, 21, or 35 and were adapted in low light for 30 min before measurements. The maximum quantum yield of PSII (Fv/Fm) was determined by a PAM fluorometer (IMAGING-PAM, Heinz Waltz, Effeltrich). Three series of experiments were set up for each culture day.

### Equipment and settings

All image acquisition tools used in this study are mentioned in Methods section. The image processing software packages using in this study are as follows: Axiovision Rel. 4.6. (Zeiss, Oberkochen), ZEISS Zen 2.6 blue (Zeiss, Oberkochen), ImageSP 1.2.9.27 (SYS-PROG & TrS, Moorenweis), BioDocAnalyze (Biometra, Göttingen), ImagingWin (Heinz Waltz, Effeltrich), Adobe Photoshop CS6 (Adobe, San Jose), Affinity Designer (Affinity, Chicago), FigTree 1.3.1, MEGA 5.2.2.

## Supplementary information

Supplementary Information

## Data Availability

Genomic data from *Durinskia capensis*, *Nitzschia inconspicua* (IRTA-CC-1), *N*. cf. *agnita* (IRTA-CC-152), *Nitzschia* sp. (SZCZP1124) and *Psammodictyon* sp. (SZCZP1020) have been deposited in NCBI under Accession Numbers LC385875-79 and LC482715.
