# Supplementary material for: Discovery of a kleptoplastic ‘dinotom’ dinoflagellate and the unique nuclear dynamics of converting kleptoplastids to permanent plastids
Source: Sci Rep. 2019 Jul 19;9:10474. doi: 10.1038/s41598-019-46852-y (PMC6642167; doi:10.1038/s41598-019-46852-y)
Supplement: Supplementary file 1 — Supplementary Information [file 41598_2019_46852_MOESM1_ESM.pdf]

Supplementary data

Title: Discovery of a kleptoplastic 'dinotom' dinoflagellate and the unique nuclear dynamics of converting kleptoplastids to permanent plastids

Authors: Norico Yamada[1*], John J. Bolton[2], Rosa Trobajo[3], David G. Mann[3,4], Przemysław Dąbek[5], Andrzej Witkowski[5], Ryo Onuma[6], Takeo Horiguchi[7] & Peter G. Kroth[1]

*Corresponding author

Author Affiliation:

1: Department of Biology, University of Konstanz, Konstanz, Baden-Württemberg, 78457, Germany

2: Department of Biological Sciences, University of Cape Town, Cape Town, Western Cape, 7701, South Africa

3: Marine and Continental Waters Program, Institute for Food and Agricultural Research and Technology, Sant Carles de la Ràpita, Catalonia, 43540, Spain

4: Royal Botanic Garden Edinburgh, Edinburgh, Scotland, EH5 3LR, United Kingdom

5: Institute of Marine and Coastal Sciences, University of Szczecin, Szczecin, West Pomerania, 70383, Poland

6: Department of Gene Function and Phenomics, National Institute of Genetics, Mishima, Sizuoka, 4118540, Japan

7:Department of Biological Sciences, Hokkaido University, Sapporo, Hokkaido,

0600810, Japan

Supplementary methods

Scanning electron microscopy (SEM)

Diatom cells were cleaned by heating with nitric acid. After washing to remove spent acid, suspensions of cleaned diatoms were dried onto 13-mm diameter round cover-slips, which were then attached to aluminium stubs by carbon discs, with silver dag painted around the edges to promote conduction. Stubs were coated with platinum for 1 to 2 min in an Emitech K575X sputter coater and examined using a LEO Supra 55VP Field Emission SEM operated at 5 kV (c. 4 mm working distance; aperture 20 μm).

DNA extraction and PCR amplification

DNA extractions of *Durinskia capensis* were performed using the QuickExtract FFPE RNA Extraction Kit (Epicentre, Wisconsin). Ten dinoflagellate cells at day 7 were isolated by capillary pipettes, and transferred into QuickExtract FFPE solution. The solution was heated to and maintained at 56 ºC for 1 hour, followed by 98 ºC for 2 min after which it was used as template DNAs without dilution. The DNAs of four diatom cultures, three *Nitzschia* spp. and *Psammodictyon* sp., were extracted by nexttec 1-step DNA isolation kit for Tissue and Cells (Biozym, Oldendorf). Two genes, the nuclear-encoded 18S rDNA of the host dinoflagellate, and the plastid-encoded *rbc*L gene from ODPs of *D. capensis* and from the above four diatoms, were amplified using the following protocol. An initial cycle at 95 ºC for 3 min, followed by 40 cycles of the

PCR steps: denaturation at 98 ºC for 20 s, annealing at 52 ºC for 15 s, and extension at 72ºC for 1 min. The final extension cycle was at 72 ºC for 1 min. The primers were the same as those used in a previous study[34]. The PCR products were purified using Gene clean Kit (Applied Biosystems, Foster City). The sequence reactions were conducted by Microsynth AG.

Molecular phylogeny analysis

The OTU sequences were aligned by ClustalW in MEGA 5.2.2[62], and then analysed by the maximum likelihood (ML) method using PhyML 3.0 beta version[63] with bootstrap analysis (1000 replicates). The selected model for ML analysis by the Akaike Information Criterion was the GTR+G (for 18S rDNA of host dinoflagellates), or GTR+G+I (for the *rbc*L gene of the diatoms). A heuristic search was performed using a SPR algorithm and the BIONJ tree[64] as the starting tree.

Supplementary references

62) Tamura, K. *et al.* MEGA5: molecular evolutionary genetics analysis using maximum likelihood, evolutionary distance, and maximum parsimony methods. *Mol. Biol. Evol.* **28,** 2731-2739 (2011).

63) Guindon, S. *et al.* New algorithms and methods to estimate Maximum-Likelihood phylogenies: Assessing the performance of PhyML 3.0. *Syst. Biol.* **59,** 307-321 (2010).

64) Gascuel, O. BIONJ: an improved version of the NJ algorithm based on a simple

model of sequence data. *Mol. Biol. Evol.* **14,** 685-695 (1997).

Supplementary table

Supplementary Table S1 / Diatom species in each dinotom species.

Group number	Dinotom species	Closest affiliation of diatom-derived plastids
**Marine *Nitzschia*-type dinotoms**		
Group 1	*Durinskia baltica*	*Nitzschia palea*
Group 2	*Durinskia* cf. *baltica*	*Nitzschia communi*s-like species 1
Group 3	*Durinskia oculata*	*Nitzschia communi*s-like species 2
Group 4	*Durinskia capensis*	*Nitzschia* cf. *agnita*<br>A close relative of *N.* cf. *agnita*
Group 5	*Durinskia kwazulunatalensis*	*Simonsenia aveniformis*
Group 6	*Galeidinium rugatum*<br>Undescribed novel dinotom 1<br>Undescribed novel dinotom 2	*Nitzschia* sp. (strain KSA0120)
Group 7	*Kryptoperidinium foliaceum*	Unknown *Nitzschia* species
Group 8	*Kryptoperdinium triquetrum*	*Nitzschia draveillensis*-related species
**Marine *Chaetoceros*-type dinotom**		
Group 9	*Blixaea quinquecornis*	*Chaetoceros* sp.
**Freshwater dinotoms**		
Group 10	*Unruhdinium jiulongense*<br>*Unruhdinium kevei*	*Discostella* sp.
Group 11	*Unruhdinium penardii*	*Discostella nipponica*
Group 12	*Unruhdinium niei*	*Cyclostephanos* sp.
Group 13	*Unruhdinium minimum*	*Cyclotella* sp.
**No genetic data of the diatom-derived plastids**		
Group 14	*Dinothrix paradoxa*<br>*Durinskia agilis*<br>*Gymnodinium quadrilobatum*	no data

Supplementary figures and the figure legends

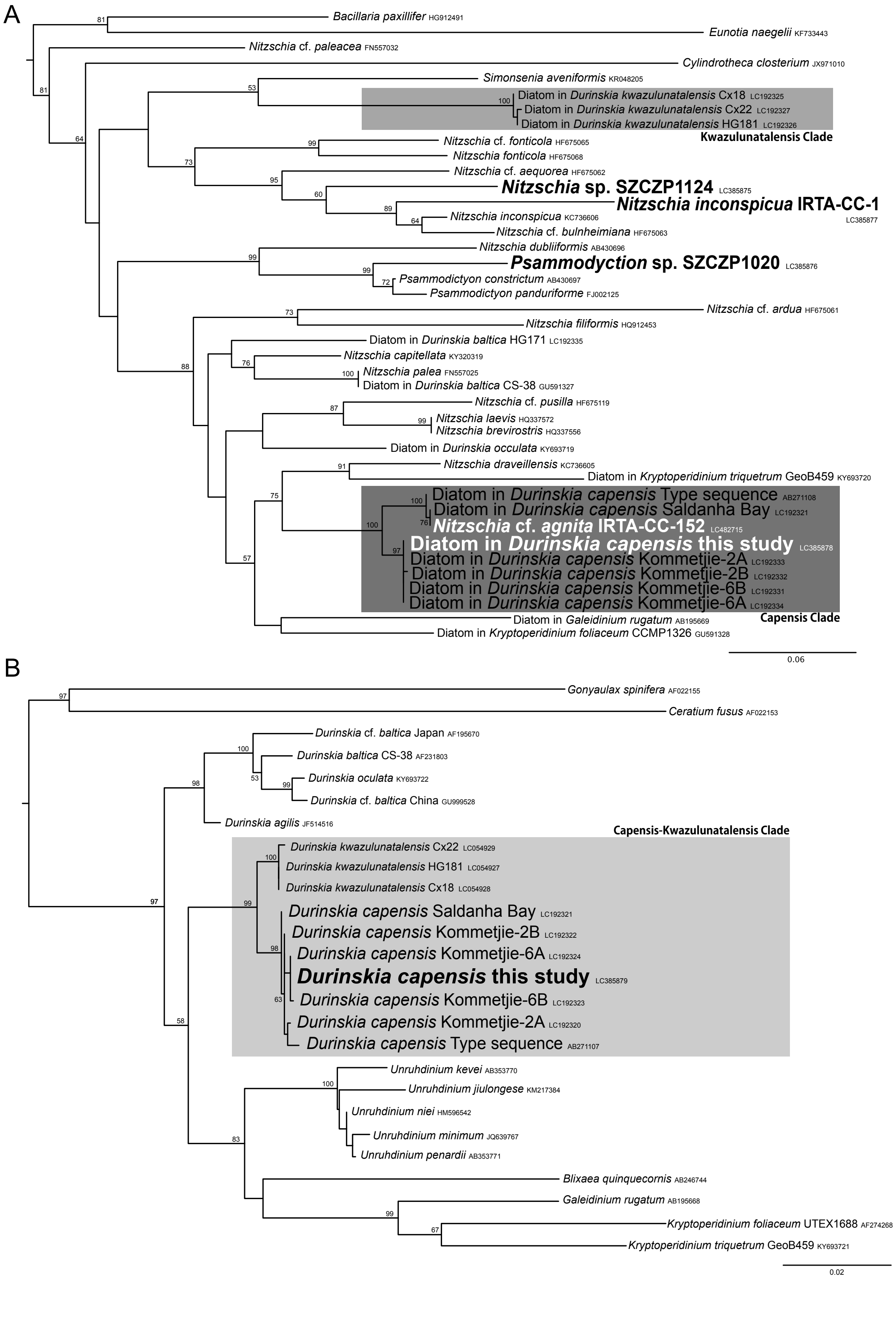

Supplementary Figure S1 / Phylogenetic reconstruction of diatoms and dinoflagellates. (A) ML phylogenetic tree of diatoms based on the *rbc*L gene. *Bacillaria paxillifer* and *Eunotia naegelii* were used for outgroups. The *rbc*L alignment comprised 1383 bp including gaps. Bold type indicates the diatoms of *D. capensis*, used in this study, and four free-living South African diatoms, *Nitzschia inconspicua* (strain IRTA-CC-1), *N.* cf. *agnita* (IRTA-CC-152) *Nitzschia* sp. (strain SZCZP1124) and *Psammodictyon* sp. (strain SZCZP1020). (B) ML phylogenetic tree based on the 18S rDNA of dinoflagellates. *Gonyaulax spinifera* and *Ceratium fusus* were used as outgroups. The 18S rDNA alignment comprised 1759 bp including gaps. Bold type indicates the *D. capensis* used in this study. In both trees, numbers on the major nodes represent ML (1000 pseudoreplicates) BS values, and only bootstrap values >50% are shown. GenBank accession numbers follow taxon names.

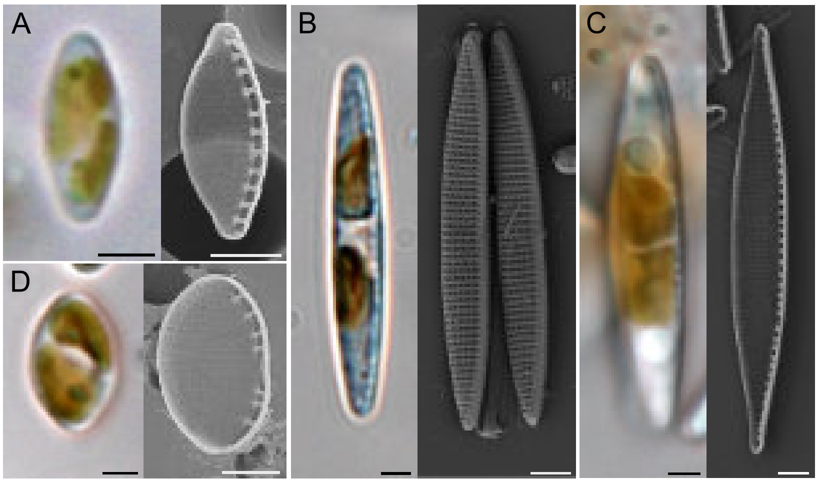

Supplementary Figure S2 / LM and SEM photos of South African *Nitzschia sensu lato* diatoms, used for feeding experiments of *Durinskia capensis*. (A) *Nitzschia* sp. (strain SZCZP1124). (B) *N. inconspicua* (strain IRTA-CC-1). (C) *N.* cf. *agnita* (strain IRTA-CC-152). (D) *Psammodictyon* sp. (strain SZCZP1020). Scale bar = 2 µm.

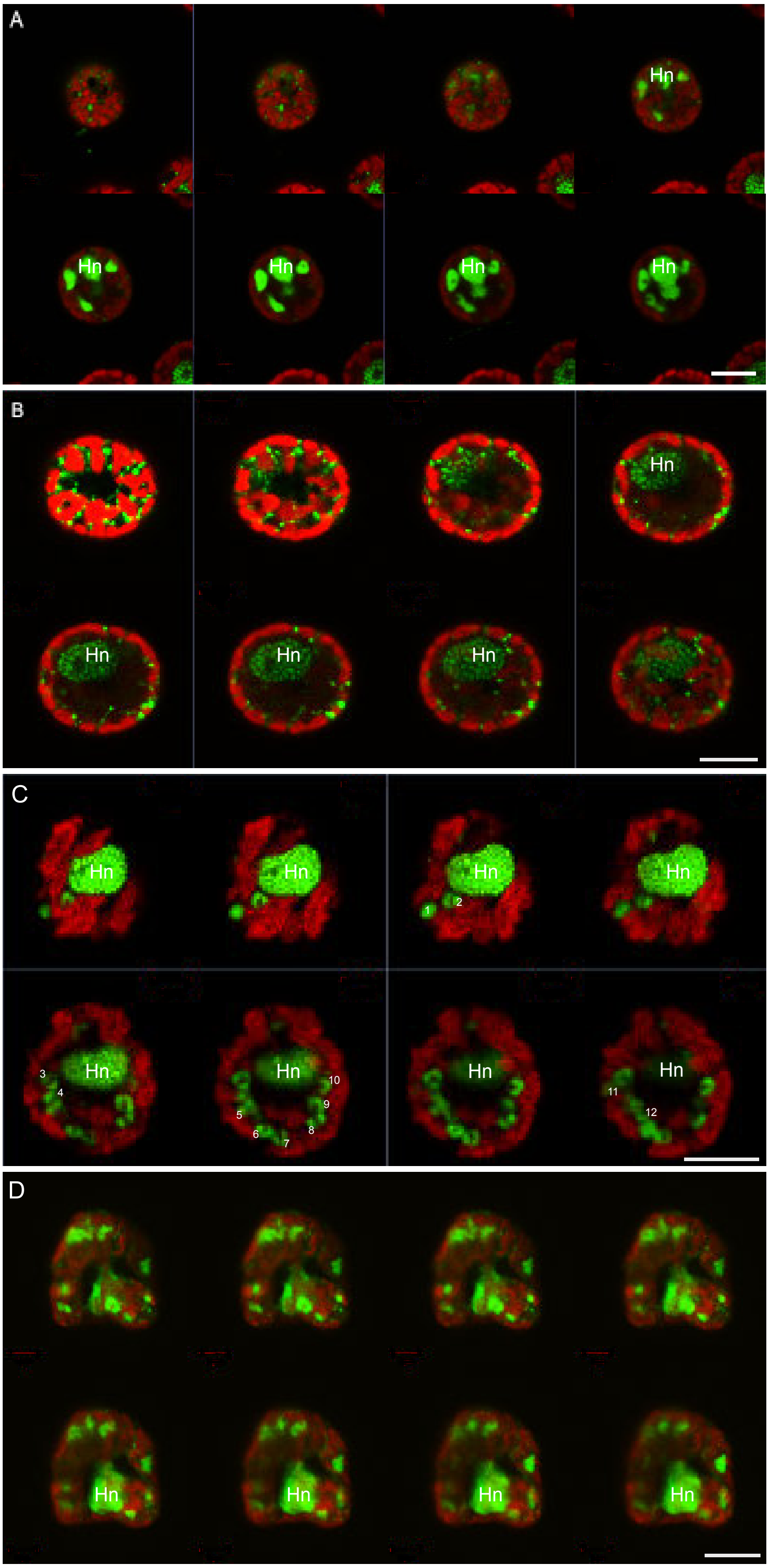

A
Hn
Hn
Hn
Hn
Hn
B
Hn
Hn
Hn
Hn
C
Hn
Hn
Hn
Hn
1
2
Hn
Hn
Hn
Hn
3
4
5
6
7
8
9
10
11
12
D
Hn
Hn
Hn
Hn

Supplementary Figure S3 / Z-stack serial photos of Figure 5, in *Durinskia kwazulunatalensis* with CLSM. SYBR-Green stained nucleus = Green, Chlorophyll *a* autofluorescence = Red. (A) A cell after the cell division. Three condensed diatom nuclei start decondensing into string or dot-shaped nuclei. Hn = Dinoflagellate nucleus. (B) A cell during interphase. (C) A cell prior to cell division, containing at least twelve condensed diatom nuclei, which indicated with numbers. (D) Early fission of the host cell. Scale bar = 10 µm.
